# Enspyre: a novel enrichment technology for selected DNA variants using pyrophosphorolysis

**DOI:** 10.1093/nar/gkaf910

**Published:** 2025-09-17

**Authors:** Katarzyna A Anton, Timon Heide, Paulina K Powalowska-Pickton, Ernesto Lowy-Gallego, Amy Lovell, Simonetta Andreazza, Efthimia Christoforou, Jeffrey Gregg, Sophie Hackinger, Magdalena Stolarek-Januszkiewicz, Robert J Osborne, Barnaby W Balmforth

**Affiliations:** Biofidelity Ltd., Cambridge CB4 0WN, United Kingdom; Biofidelity Ltd., Cambridge CB4 0WN, United Kingdom; Biofidelity Ltd., Cambridge CB4 0WN, United Kingdom; Biofidelity Ltd., Cambridge CB4 0WN, United Kingdom; Biofidelity Ltd., Cambridge CB4 0WN, United Kingdom; Biofidelity Ltd., Cambridge CB4 0WN, United Kingdom; Biofidelity Ltd., Cambridge CB4 0WN, United Kingdom; Biofidelity Inc., Morrisville, NC 27560, United States; Biofidelity Ltd., Cambridge CB4 0WN, United Kingdom; Biofidelity Ltd., Cambridge CB4 0WN, United Kingdom; Biofidelity Ltd., Cambridge CB4 0WN, United Kingdom; Biofidelity Ltd., Cambridge CB4 0WN, United Kingdom

## Abstract

Hybridization capture has been a mainstay of molecular enrichment technologies over the last 15 years, providing robust enrichment of target molecules. However, it typically requires significant sequencing depth to capture rare variants, which limits its efficiency and increases costs, especially in applications like minimal residual disease (MRD) monitoring and early cancer detection. Enspyre (Enrichment by selective pyrophosphorolysis and release) is a novel technology designed to address these limitations by enabling selective enrichment of specific variants prior to sequencing. Here, we present proof-of-concept showing that Enspyre can enrich specific variants by a mean of 35-fold compared to hybridization capture. To demonstrate Enspyre’s utility in oncology, we tested serial dilutions of contrived MRD samples using 1800 custom-designed Enspyre bait probes. Our results show that Enspyre accurately detects and quantifies the presence of MRD down to 0.01%–0.001% VAF (variant allele frequency) or 10–100 parts per million (ppm), achieving this sensitivity with approximately 4% of the sequencing reads. Overall, Enspyre has the potential to support applications in rare variant detection, early cancer detection, and therapy selection with reduced sequencing depth, data processing time, and storage requirements.

## Graphical Abstract

**Graphical Abstract:**
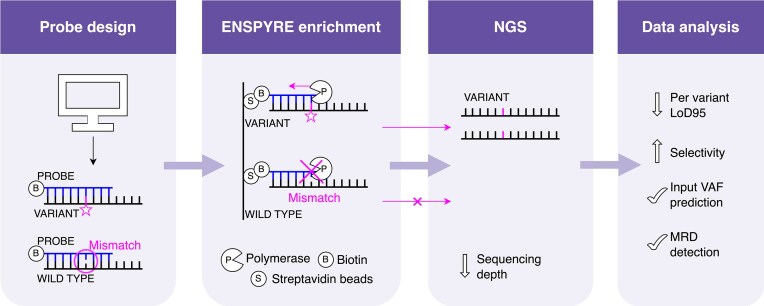

## Introduction

Next-generation sequencing (NGS) technology has significantly advanced DNA-related research, influencing fields from ancient genome studies and agriculture to molecular diagnostics. However, the cost of performing whole-genome sequencing (WGS) at the required depth to detect rare variants remains a challenge, particularly in clinical applications. As a result, several strategies have been developed to obtain necessary genomic information with minimal sequencing. One of the most effective solutions is hybridization capture, which efficiently targets and enriches specific genomic regions within complex DNA samples using target-specific probes. This approach is particularly useful for fragmented nucleic acid samples, such as ancient DNA or DNA extracted from formalin-fixed paraffin-embedded (FFPE) tissue, where polymerase chain reaction (PCR) amplification may not succeed due to incomplete sequences [1].

Hybridization capture is widely used in applications such as whole-exome sequencing [2], detection of genetic diseases and cancer mutations [3], infectious disease characterization [4], and population studies [5]. Despite its success, this method has limitations, especially in detecting rare variants. Since it enriches broader genomic regions rather than specific variants, distinguishing rare alterations from background noise can be challenging. In oncology, where the detection of rare variants is critical for diagnosis and guiding therapeutic decisions, more precise tools are required. For instance, in liquid biopsy, clinically important variants are present at very low levels [e.g. 0.01%–0.0001% variant allele frequency (VAF)], and high-sequencing depths are required to identify them, resulting in increased costs, time, and enormous volumes of data that must be stored, analysed, and transferred.

Enspyre (Enrichment by selective pyrophosphorolysis and release) is a novel target enrichment technology designed to address these challenges. Leveraging the high specificity of the pyrophosphorolysis reaction to double-stranded DNA, Enspyre selectively enriches specific variants within targeted regions. Through this enrichment, Enspyre minimizes background noise and increases the fraction of variant-containing sequence reads, allowing rare variants to be detected at lower sequencing depths.

Minimal residual disease (MRD) refers to the small number of cancer cells that remain after treatment. Their detection is critical for monitoring patient response to therapy and predicting recurrence, directly impacting clinical decision making. Current NGS-based MRD tests are often tumour-informed, meaning they require a patient’s tumour sample to identify their specific variants to track over time. While PCR-based MRD tests can track a limited number of variants (up to 50–100), hybridization capture methods can track as many as 1800 or more patient-specific variants [6]. A higher number of targeted variants improves the likelihood of detecting cancer-related alterations and overall assay sensitivity. However, this approach generates much larger volumes of sequence data, which drives up costs and increases analysis time, making it less practical for scaling to widespread clinical use.

Enspyre offers an alternative: tracking of a large number of variants with significantly reduced sequencing depth. This lowers costs and enables the use of low-throughput sequencing platforms with the potential to make MRD detection feasible in smaller clinical laboratories.

In this paper, we present Enspyre proof-of-concept and explore its applicability in MRD detection.

## Materials and methods

### DNA fragmentation

Samples used to generate the technology proof-of-concept data were Oncospan cell-free DNA (cfDNA, Horizon, HD833) and SeraCare Custom GM24385 WT ctDNA v4 mix (SeraCare, 0710-2640).

HG002 and HG003 DNA samples were obtained from the NIGMS Human Genetic Cell Repository at the Coriell Institute for Medical Research. HG002 and HG003 DNA samples were fragmented by sonication using S220 Focused-Ultrasonicator (Covaris) to a fragment size distribution of ∼150 bp to match that of ctDNA (circulating tumour DNA). Fragment size distributions were confirmed using 4150 TapeStation System (Agilent) with High sensitivity D1000 screentape (Agilent, 5067-5584) and reagents (Agilent, 5067-5585). Sample concentration was verified by Qubit Flex Fluorometer (Invitrogen) with dsDNA Quantitation High Sensitivity Kit (Invitrogen, Q33230).

### Contrived sample preparation

Contrived DNA samples for the technology proof-of-concept were prepared by diluting Oncospan cfDNA 1:10 in Seracare ctDNA WT background. Contrived DNA samples for the MRD proof-of-concept were produced by serially diluting “mutant” HG002 DNA into “wild-type” HG003 DNA. Dilutions ranged from 10% VAF concentrations of the heterozygous alternate alleles (targeted unique variant alleles) down to 0.0001% concentrations.

### Identification of suitable exonic variants in HG002, HG003, and probe design

For the MRD proof-of-concept, we identified variants [single-nucleotide variants (SNVs); insertions and deletions (InDels)] that were heterozygous in the Personal Genome Project [7] (PGP)—Family 3140—Son (proband) HG002 (NA24385) and absent in the Personal Genome Project—Family 3140—Father HG003 (NA24149) by filtering whole genome sequencing data from corresponding cell lines (sourced from the Coriell Institute). To enable this, we obtained variant calls produced by the Genome in a Bottle (GIAB) consortium from NIST v4.2.1 [8] and filtered them using BCFtools [9]. This approach identified a total of 404 299 high-confidence variants present in HG002 but not HG003. We further refined the selection by filtering variants located within 100 bp of target regions defined by the 35.1 Mb SureSelectXT Human All Exon V8 sequencing panel. After this filtering, a total of 12 673 variants remained.

### Panel design and probe synthesis

To generate the technology proof-of-concept data we used a fixed panel probe pool targeting 2348 clinically relevant variants, such as the SNVs and InDels present in Oncospan cfDNA (Horizon, HD833), Acrometrix Oncology HotSpot Control 5% (Fisher Scientific, 15 902 951) samples, and Biofidelity’s Aspyre Lung [10]. These probes were designed with a simplified set of rules primarily based on the melting temperature of probes without the machine learning (ML) model described below.

For the MRD proof-of-concept, we designed a first panel (Pool 1) that selected variants at random from the entire set of 12 673 filtered down HG002 variants and a second panel (Pool 2) that only selected SNVs and InDels with a predicted probe performance score (obtained from the probe design tool) above the respective median score for each variant type (InDels: > 85.0, SNVs: > 210). For each panel, 1800 variants were selected, of which 1620 (90%) were targeting SNVs and 180 (10%) targeting InDels.

The distribution of SNV substitution types and InDels composing our two probe sets was randomly selected to mirror the somatic variant distribution observed in lung cancer, as described by Lawrence *et al.* [11] and Priestley *et al.* [12].

Insertions/deletions with length >40 bp were removed due to the difficulty of resolving them using short read sequencing. We then used the same sampling approach to select a final set of InDels with equal proportions of single deletions (SDEL), single insertions (SINS), multiple deletions (MDEL), and multiple insertions (MINS).

The length of the designed probes varied between 25 and 138 bases (mean 60 bases) for Pool 1 and between 24 and 91 bases (mean 53 bases) for Pool 2. The variant position was on average 7 bases ± 4 bases away from the 3′ end of the probe. The melting temperature of the hybridizing regions of the probes was set to the minimum of 65°C. The pools of probes were ordered from Integrated DNA Technologies and captured onto beads via biotin–streptavidin bond.

Each probe pool was combined at equimolar concentration with a fixed pool of 2199 probes as well as five control probes used for internal quality control (QC) of samples. Control probes were designed against selected germline variants which return comparable molecule numbers for each run and can be used for normalization and identification of failed samples (i.e. with <100 molecules returned for at least one control probe). This process allowed us to demonstrate the versatility of Enspyre regarding combining different panels and comparing data sets between different optimization experiments.

### Training of the probe design tool

For the MRD proof-of-concept experiments, a probe for each of the targeted variants was designed using our proprietary in-house probe design algorithm predicting probe performance. This algorithm is based on the RandomForestRegressor ML model trained using NGS datasets with random variation of different probe features. For training the ML model, three sequencing datasets were generated from 48 GIAB samples (HG002 in HG003 at 10% VAF) using 5450 unique probes (three probe pools). A wide variety of probe features were used as input for training, such as melting temperature, lengths of the probe sequences around the variant, and GC content. The target variable used in the model training was derived by modelling the expected alternate count using a Poisson regression on the experimental data. This model accounts for the performance of each probe, the input VAF, and sample-to-sample variability. Comparing the predicted to the actual probe performance yielded an *r*^2^ value of 0.6847. This score could not be improved further with the current set of probe features and the quality of synthesized probe pools.

For each of the considered variants (SNVs and InDels <40 bp), our probe design tool generated a range of designs with varying lengths of complementary regions upstream and downstream of the variants. This process ensured that all possible combinations within the desired range were covered. For each design, the melting temperature was calculated, and only designs with a temperature ≥65°C were selected. Next sequence-based features were calculated and fed into our RandomForestRegressor ML model. This model predicts the performance of each probe and assigns a score accordingly. Probes with higher predicted performance scores are generally more effective at retrieving alternate molecules. Finally, the tool selected the design with the highest predicted performance score as the probe for a particular variant.

### Limit-of-detection experimental setup

Limit-of-detection (LoD) data were generated on contrived samples with allele fractions ranging from 0.0001% to 10% VAF. Four 0% VAF controls for each probe set were processed in parallel with these samples. Each input MRD level was tested in two technical replicates by two operators (i.e. four replicates in total) with both probe pools. This amounted to two separate Enspyre and sequencing runs, 28 samples per run. Twenty-eight additional HG003 samples were processed to generate Limit of Blank (LoB) data for each probe pool (two Enspyre and sequencing runs).

To establish linearity of molecule recovery with input in Enspyre (Figs 4A and Supplementary Fig. S7A), an additional run was performed using only Pool2 and samples at intermediate VAFs: 1%–10% and 0% VAF controls (24 samples and 4 replicates each).

### Pre-Enspyre library preparation

Library preparation for the technology proof-of-concept experiments were produced according to the manufacturer’s protocol with NEBNext^®^ Ultra^™^ II DNA Library Prep Kit for Illumina (New England Biolabs, E7645L) from a total of 20 ng of input DNA per sample.

Library preparations for the MRD proof-of-concept experiments were produced according to the manufacturer’s protocol with Watchmaker Genomics DNA Library Preparation Kit (7K0103-096) from a total of 20 ng of input DNA per sample.

All adapter-tagged libraries were amplified in an 8-cycle PCR reaction. xGen^™^ CS adapter (IDT, 1080799) as well as xGen^™^ UDI Primer Plate 1, 8 nt (IDT, 10 μM, 10005922) were used in all the pre-Enspyre library preparation protocols.

### Non-selective target enrichment

Oncospan cfDNA (Horizon, HD833) samples were diluted 1 in 100 in SeraCare Seraseq WT ctDNA and processed using NEBNext^®^ Ultra^™^ II DNA Library Prep Kit for Illumina (New England Biolabs, E7645L) followed by SureSelect Cancer All-In-One Solid Tumor, 16 rxn, index 1–16 hybrid capture kit (Agilent, G9704S) according to manufacturers’ protocols. Samples were pooled at equimolar quantities and sequenced together with Enspyre-processed samples using a mid output kit for 2 × 150 cycles to produce paired-end reads by the Cambridge Biochemistry DNA Sequencing Facility (University of Cambridge).

### Enspyre target enrichment

Adapter tagged libraries (see “Pre-Enspyre library preparation”) were subjected to target enrichment using the Enspyre protocol. First overnight (16 h) hybridization was performed using custom probe pools (IDT; concentration of 2 pM per probe) and the input of 900 ng per sample (300 ng for technology proof-of-concept studies presented in Fig. 2C). The hybridization buffer used was Invitrogen^™^ ULTRAhyb^™^ Ultrasensitive Hybridization Buffer (Fisher Scientific, 10792117). Proprietary biotinylated oligo (commercially available Biofidelity Ltd.) was added to capture the probes onto beads via biotin–streptavidin bond. Additional blockers used: COT-1 DNA (Fisher Scientific, 11568726), salmon sperm DNA (Fisher Scientific, 10605543), and xGen Universal Blockers TS (IDT, 1075475). Following hybridization, hybridized molecules were captured on 10 μl of Dynabeads MyOne Streptavidin C1 (Thermo Fisher Scientific, 65002) per sample in a bead binding buffer [13]. Beads were then washed three times to remove off-target molecules with a 100 μl wash buffer 1 (wash buffer 2 in Moss *et al.* [14]) at 70°C for 5 min each time. An additional wash was carried out using 100 μl wash buffer 2 (commercially available Biofidelity Ltd.). Biofidelity’s proprietary pyrophosphorolysis (PPL) reaction was set up directly on the washed beads using 20 μl of PPL mix (commercially available Biofidelity Ltd.) at 45°C for 5 min, followed by a 60°C release step. Supernatant was then collected while beads remained on the magnet and released molecules amplified in 50 μl volume using Equinox PCR mastermix (Watchmaker Genomix, 3K0014-50ML) with 0.5 mM p5/p7 primers following the manufacturer’s protocol. The reaction was purified using SPRIselect beads following manufacturer’s instructions with a bead to sample ratio of 0.9× (Beckman Coulter, B23319). Sample concentrations were quantified via Qubit Flex Fluorometer (1× dsDNA High Sensitivity Kit, Invitrogen, Q33230) and qPCR (KAPA Library Quantification Kit, Roche, 7960140001) using CFX384 Touch Real-Time PCR Detection System (BioRad). Fragment sizes were measured using Tapestation (High sensitivity D1000 screentape and reagents, Agilent, 5067-5584 and 5067-5585).

### Sequencing

DNA from 28 samples (32 samples for Fig. 2C) was pooled in equimolar quantities to produce final sequencing libraries. The pool was loaded at a concentration of 2 pM onto a NextSeq 500 sequencer (Illumina) and sequenced with a mid output kit for 2 × 150 cycles to produce paired-end reads by the Cambridge Biochemistry DNA Sequencing Facility (University of Cambridge).

### Sequencing data analysis

Sequencing data were processed with an in-house developed analysis pipeline to quantify the number of duplex and non-duplex molecules for each variant. In brief, reads were aligned against the human reference genome using Burrows-Wheeler Aligner [15] (BWA-MEM) and grouped into read bundles containing reads with identical breakpoints and similar UMI sequences with a specific edit distance for collapsing of $ \le$2. Consensus variant calls were derived for all targeted variants that overlapped a read bundle. For each variant the number of variant read bundles that had a consensus supported by both original input strands were counted to determine duplex (variant) molecule counts. The number of read bundles where variants were only supported by reads of one original input strand were counted to determine the number of “single strand molecules.” The sum of both molecule types is the total number of variant molecules. Counts for wild-type molecules, in which no variant of interest was supported, were determined in the same way.

### Assessment of assay selectivity

The selectivity metric $S = \frac{{( {1 - f} )m}}{{fw}}$ was calculated, where $m$ denotes the number of recovered variant molecules, $w$ the the number of recovered wild-type molecules, and $f$ the VAF of the variant alleles in the input DNA for each probe/sample pair. Next this value was averaged across all samples with an input VAF of at least 0.1% for each probe to determine the probe level selectivity value.

The *S* value was also calculated for data aggregated across all probes to determine the global (i.e. cross-panel) selectivity ${{S}_g}$. Alternatively, this value can be expressed as the weighted harmonic mean of the per-probe selectivity values $S$ where each probe is weighted by the fraction of input molecules ${{x}_{m,i}}$ the probe recovers on average.

To determine the relative sequencing amount ${{c}_r}$ of Enspyre compared to a conventional sequencing assay with identical off-target ratio one can calculate ${{c}_r} = \frac{{1/{{f}_o}}}{{1/{{f}_i}}} = \frac{{fi}}{{fo}} = \frac{1}{{E{{F}_{{{f}_i}}}}}$, where $E{{F}_{{{f}_i}}}$ is the enrichment factor (i.e. the ratio of output ${{f}_o}$ over input ${{f}_i}$ VAF) that can be determined as $EF{{f}_i} = \frac{{fo}}{{fi}} = \frac{{{{S}_g}}}{{1 - {{f}_i} + {{f}_i}{{S}_g}}}$, where ${{f}_i}$ is the VAF at which we want to determine the relative sequencing amount required and ${{S}_g}$ the global (i.e. cross-panel) selectivity.

### Calculation of assay LoD

LoD of the assay is defined as the input VAF at which 95% of samples (LoD95) are called with a threshold strict enough to meet a false-positive rate (FPR) that would correspond to less than one expected false-positive variant call in 160 samples (i.e. FPR < 2.84 × 10^−6^ for each variant). To estimate LoD, we used a Poisson regression model of the Enspyre assay considering the performance of individual probes, the input VAF, as well as sample-to-sample variability. We then applied this model to predict LoD95 for all the targeted variants.

### Calculation of background error rates

Two types of priors for the background error rates of the molecule counts per site were considered: firstly, a prior for all variant sites of a given substitution type and secondly, a prior for each variant site (i.e. a site-specific prior).

For the first prior, per-site error rates were estimated for each probe p as $\lambda_{e,p}^{\wedge} = \frac{1}{N}\mathop \sum \limits_{i = 0}^N {{x}_i}$ across all samples $i$. Next, the estimation of moment matching parameters ɑ and β for a Gamma distribution on all probes of a given substitution type was performed as $\alpha = \frac{{{{\mu }^2}}}{{{{\sigma }^2}}}$ and $\beta = \frac{\mu }{{{{\sigma }^2}}}$, where $\mu$ is the mean of the per-site error rate of all probes of a given substitution type and $\sigma$ the variance of the per-site error rates across all probes of a given substitution type.

For the site-specific prior $\alpha = 0.1 + \frac{1}{N}\mathop \sum \limits_{i = 0}^N {{x}_i}$ and $\beta = 1 + N$ were obtained as prior for the per-site error rates of the MRD model. This is the Gamma posterior given the prior $P( {{{\lambda }_e}} ) = Gamma( {\alpha = 0.1,\ \beta = 1} )$ and assuming ${{x}_i}\sim Poisson( {{{\lambda }_e}} )$ for the data of each site.

### Identification of contaminated samples

LoB is defined as the highest apparent analyte concentration expected to be found when replicates of a blank sample containing no analyte are tested. During LoB determination, we identified a subset of samples that appeared to have cross-contamination, evidenced through manual analysis of Genome in a Bottle (GIAB) linked SNPs (within 100 bp of the original variant). We used the ratio of the number of molecules with both SNPs present to the number of molecules with the original variant and the site of the second variant as an approximation of all identified duplex variants derived from contamination.

### Input VAF inference and variant calling

Input VAF levels were called using a proof-of-principle variant calling model for Enspyre. To this end, the model to call MRD was modified to obtain probe performance parameters from the data of 10% input VAF samples (i.e. parameter inference given the real input VAF). The parameterized model was then used to predict per-variant input VAF levels from the data. To avoid overfitting to a specific sample, the model performance was assessed using nested cross-validation.

### MRD calling

MRD levels were called using an in-house hybrid model combining the probe design scoring model with a mechanistic Bayesian model of the assay. This model is composed of two parts: a background error model based on observed error rates for different substitution types and a model describing the expected additional signal at different MRD levels for each probe. The variability across probes is determined based on the predicted probe scores. Sample level variability of the assay’s performance are determined from: control probes, the input mass into library preparation, and the input mass into target capture. The information of non-duplex and duplex calls for each probe is integrated across all probes to determine an overall probability of MRD and the MRD level for a sample.

## Results

### Enspyre is a new technology that allows for enrichment of specific variants prior to sequencing

**Figure 1.**
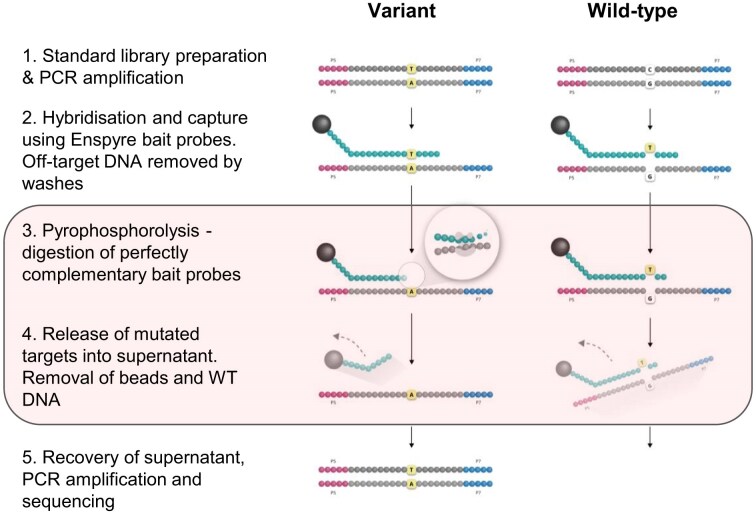
Enspyre enables selective enrichment of selected variants for NGS. Enspyre is a modified hybridization-capture approach. DNA is initially processed using a standard library preparation kit with an adapter and Dual UMI indexing primers. Next, a custom pool of probes hybridizes to the library. Hybridized molecules are captured on streptavidin beads and off-target DNA is removed by washes. Steps 3 and 4 (framed) involve pyrophosphorolysis where an exonucleolytic polymerase activity is used to digest probes complementary to the targets. As probes are designed to recognize variants, only these hybridized to mutated molecules are fully digested and the hybridized molecules released to the supernatant. Supernatant with variants is then collected, amplified by PCR, and sequenced.

The Enspyre technology enriches rare variants prior to detection by applying a molecular biology solution we developed at Biofidelity [16] to a traditional hybridization capture workflow (Fig. 1). The innovation consists of an added step of pyrophosphorolysis performed directly on molecules captured by target-specific probes on streptavidin beads. Pyrophosphorolysis is based on a reverse polymerase reaction mediated by a DNA polymerase in the presence of pyrophosphate ions (PPi) and is used to digest probes that are fully complementary to targets [17]. Any mismatch will block the enzyme from further digestion and progressing along the probe (Supplementary Fig. S1). Since the probes are specifically designed to detect mutations, only those bound to selected variants are fully digested and molecules containing these variants released into the supernatant. The supernatant with liberated molecules is then collected and processed it further for sequencing.

**Figure 2.**
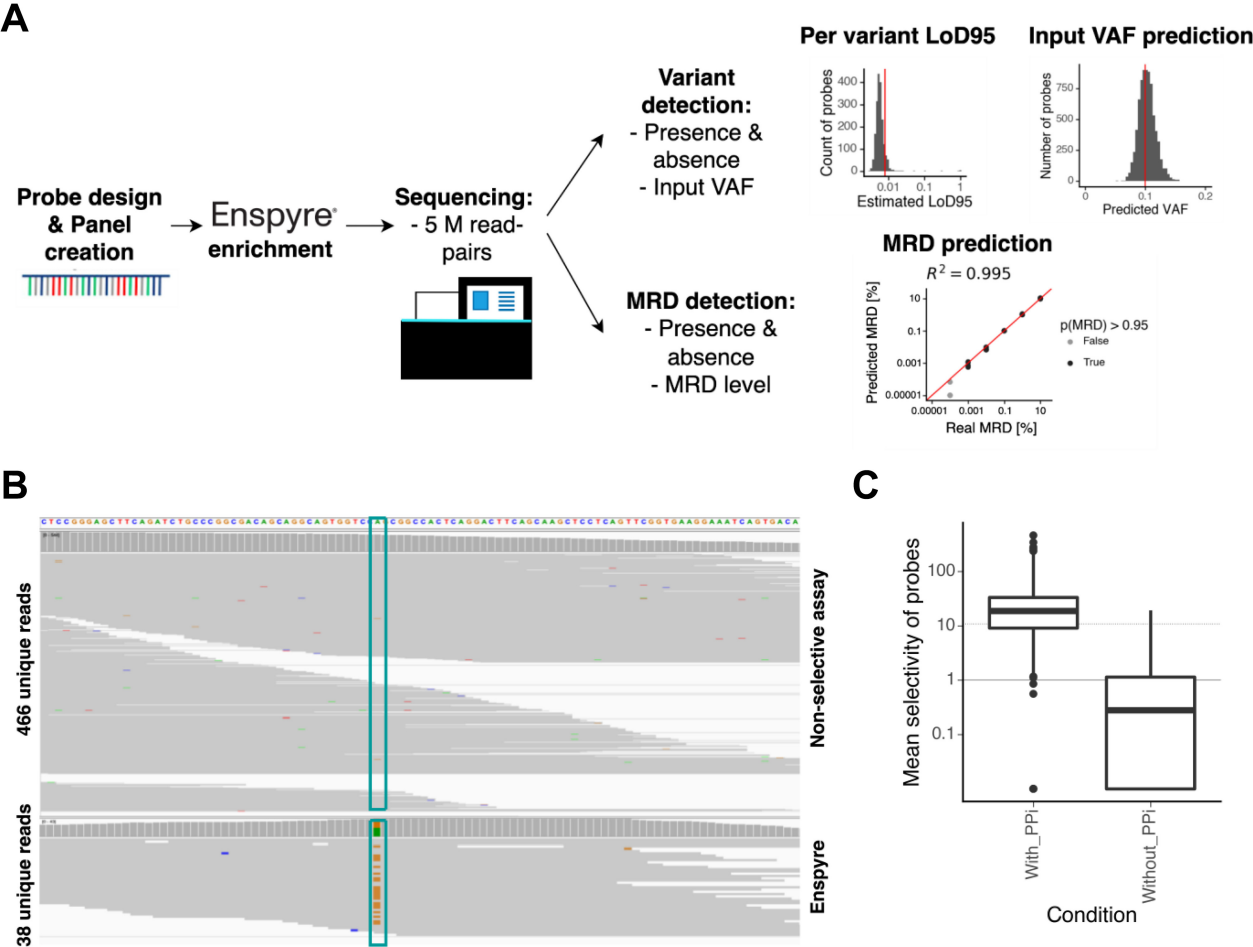
Enspyre workflow and technology proof of concept. (**A**) Visual representation of an Enspyre workflow. Firstly, variant specific probes are designed. Enspyre hybridization-capture is performed to enrich for patient-specific variants. Following NGS, variant detection and optional MRD prediction are performed. (**B**) A screenshot of IGV view for variant rs1056171 comparing recovery of the alternate versus WT molecules using a non-selective versus Enspyre assay. (**C**) Selectivity is defined as as the ratio of the fraction of mutant input molecules over the fraction of wild-type input molecules recovered by any sequencing assay. The presence of PPi enables the PPL reaction and makes Enspyre selective for mutant molecules.

For practical applications, we built this molecular biology solution into an Enspyre workflow. In the first step, we design a variant specific probe pool (Fig. 2A). Once the probe panel is created, we subject DNA samples to standard library preparation procedures using commercially available kits, followed by hybridization capture with the predesigned, sample-specific probes (Fig. 1). We remove off-target molecules by a series of washes, followed by pyrophosphorolysis performed directly on beads. We collect the supernatant enriched for targeted molecules, amplify them via PCR and sequence using standard next generation sequencing. Post-sequencing, we analyse the data using the Enspyre analysis pipeline (Supplementary Fig. S2A), which includes QC checks, UMI-trimming, alignment to a reference genome, UMI-based error correction and estimation of molecule numbers, as well as other optional sample-specific analyses (e.g. somatic variant identification, input VAF prediction, and MRD assessment; Supplementary Fig. S2B).

The initial optimization experiments testing feasibility of Enspyre included optimization of the buffer, reaction conditions, and incubation parameters. To ensure reliable capture of targeted genomic regions and enrichment of variant-containing molecules, we used prediluted Oncospan cfDNA HD833 reference standard samples. Samples were processed through both standard hybridization capture and Enspyre workflows, enabling a rough comparison of performance. The advantage of using Enspyre over a non-selective assay is particularly well illustrated in the IGV screenshot shown in Fig. 2B demonstrating enrichment of one of the variants in our early experiments (variant rs1056171, 0.5% VAF). While a non-selective hybridization capture assay returned 2 variant molecules among 466 unique reads (0.43%), after performing Enspyre the number of variant molecules increased to 26 out of 38 unique reads (68.4%).

To demonstrate the capabilities of Enspyre, we performed a proof-of-concept experiment using a fixed panel of 2348 probes targeting various lung and colorectal cancer-associated somatic variants. Included in this set were probes specific for mutations present in the oncology reference standard Oncospan cfDNA HD833. To show that the pyrophosphorolysis step of Enspyre is selective and can be used to enrich multiple variants simultaneously, we diluted the Oncospan standard in the Seracare ctDNA WT background and subjected these samples to the Enspyre protocol with and without PPi. Without PPi, pyrophosphorolysis and the associated release and enrichment of targeted molecules should not occur [16].

To quantify the selective enrichment of alternate molecules (targeted unique variant molecules) against wild-type molecules by the assay, we derived a metric called selectivity (*S*). Selectivity is the ratio of the fraction of input alternate molecules over the fraction of input wild-type molecules that are recovered by any sequencing assay independent of the input VAF. For a non-selective assay (i.e. standard target capture), *S* will be equal to one. Values of *S* > 1 indicate enrichment for the targeted allele.

In the technology proof-of-concept experiment, mean selectivity of all the Oncospan targeting probes for the condition with PPi was 40.7 (median: 18.7), while without PPi it dropped to 1.15 (median: 0.3; equivalent of a non-selective assay; Fig. 2C). This shows that following Enspyre the fraction of alternate over wild-type molecules recovered increased by a mean of 35-fold if the PPL reaction took place. In this experiment, 36 out of 235 Oncospan-targeting probes did not return any alternate molecules, likely due to not optimized probe designs, poor oligonucleotide synthesis, or very low input VAF of some targets in the pre-diluted Oncospan samples (input VAFs ranging from 0.1% to 5%).

### Probe panels can be scaled to thousands of probes in applications such as high-sensitivity tumour-informed MRD

Following the technology proof-of-concept, we performed a set of experiments to demonstrate that Enspyre can be used to reduce the sequencing depth requirements of clinical NGS applications such as MRD detection. For this purpose, we selected contrived samples created from Genome in a Bottle (GIAB) DNA [8] as they offered a much higher number of variants to detect simultaneously compared to commercially available oncology reference standards. We sourced and sonicated two types of DNA: son and father from the same family (Coriell Institute, Personal Genome Project). We then selected a subset of heterozygotic variants present in the son (HG002) but absent from the father (HG003) (Supplementary Fig. S3A) to target and enrich via Enspyre.

Recognizing probe design as a crucial factor of successful variant enrichment, we used an ML-based probe design algorithm that selects the optimal Enspyre bait probe for a given variant. The RandomForestRegressor ML model was chosen as it offers several advantages: high predictive accuracy, robustness to overfitting, and effective handling of datasets with many features [18]. The design algorithm scores each probe based on its features. High scores for a probe mean it should recover targeted molecules more efficiently during Enspyre. Lower scores indicate lower efficiency of targeted molecule recovery.

For this study, we designed two different probe pools (Supplementary Fig. S3A). For Pool 1, we selected variants randomly, without considering the predicted performance of their probes. For Pool 2, we only sampled variants for which the probe design score was above the median (see “Materials and methods” section for details of this selection). For these variants we are generally confident that the probes should return a high number of alternate molecules and would benefit variant detection. To ensure an accurate representation of variants observed in MRD detection and oncology monitoring, we sampled different types of variants for both probe pools, so that their distribution would reflect that of lung cancers with 90% of SNVs [11] (Supplementary Fig. S3B) and 10% of InDels [12] (Supplementary Fig. S3C) and with the different types of SNVs and InDels sampled at clinically representative fractions.

We then used GIAB contrived samples with simulated MRD levels ranging from 0.0001% to 10% VAF to assess the performance of Enspyre. Each of the sequencing runs performed for this set of experiments resulted in ∼4.95 million reads per sample on average (Supplementary Fig. S4A). This number is relatively low compared to other NGS-based tumour profiling assays assessing a similar number of variants, for example NeXT Dx with 450 million reads per sample [19] or NeXT Personal with 330 million reads per sample [20] both from Personalis.

Importantly, the occasionally observed sample failures resulting in no alternate molecules (Supplementary Fig. S4B) are easily identified from an internal QC metric. In these experiments, we identified a total of 14/112 (12.5%) failed samples which were excluded from the analysis. This failure rate was likely a result of interfering substances carried through from upstream steps to the PPL reaction. Subsequent refinements to the protocol, including further optimization of the washing steps, have reduced the dropout rate in more recent experiments to ∼6%.

### Enspyre probes are designed rapidly without testing or optimization

**Figure 3.**
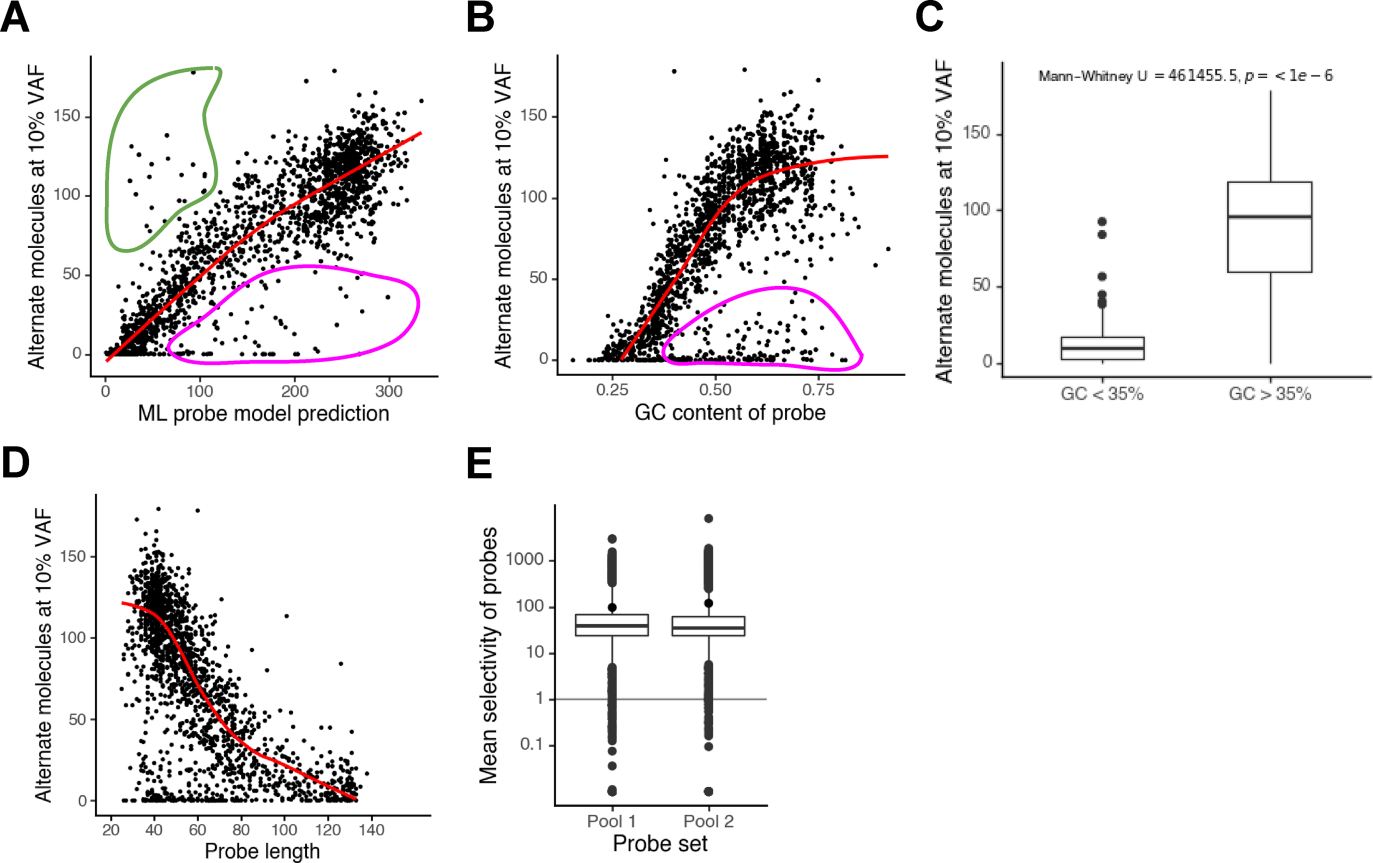
Probe design works well to predict Enspyre bait performance. (**A**) Observed mutant molecule recovery of each probe in Pool 1 at 10% input VAF plotted against the probe design algorithm scores, shows a clear correlation of the two values (Pearson’s = 0.74). While the correlation between prediction and performance is generally very good, there are some outliers: circled in magenta are probes with performance worse than expected; circled in green are probes with behaviour better than expected. (**B**) Observed mutant molecule recovery of each probe at 10% input VAF plotted against the GC content of the probe sequence. Circled in magenta are non-working probes which performance is GC content-independent. (**C**) Probes with a GC content below 35% do not work compared to probes with a GC content ≥ 35%. (**D**) Increasing length generally decreases the probe performance (potentially due to issues with synthesis). Low GC-content probes are often longer to match the melting temperature of 68°C (a requirement for Enspyre) which might explain lower recovery of these mutant molecules (B). (**E**) Selectivity values for each probe were averaged from data for samples with input VAFs ≥ 0.1%. Selectivity values were not significantly different between probe sets 1 and 2 (mean and distribution).

To test how effective the probe design was in this experiment we calculated the average number of alternate molecules recovered by each probe at the highest tested input VAF of 10%. These values for both pools were highly correlated with the probe scores predicted by the probe design algorithm (Fig. 3A and Supplementary Fig. S5A). As expected, for Pool 2 both probe design scores and the number of retrieved alternate molecules were generally higher than for Pool 1, further confirming that using the developed ML model we can design probes efficiently targeting variants of interest.

From the polynomial regression for Pool 1, we were able to estimate the assay performance for efficient probes (i.e. with a predicted score of above 200, such as included in Pool 2). These probes on average returned around 120 alternate molecules out of about 633 present in the original input into library preparation (calculated from: 20 ng library prep input, 10% VAF, 3.158 pg of haploid male DNA per cell following Dolezel *et al.* [21], GRCh38.p13 Assembly Statistics: https://www.ncbi.nlm.nih.gov/datasets/genome/GCF_000001405.39/, accessed on 3 September 2024). This amounts to 19% of alternate molecules retrieved following library preparation and Enspyre. Considering that library preparation has ∼40% efficiency (data not shown), the estimated efficiency of the Enspyre steps in these experiments was ∼47% demonstrating it could be a useful tool in rare variant detection.

To understand which types of variants are particularly challenging to target in Enspyre, we explored different probe features of Pool 1. A key feature identified here was the GC content of the probe sequence (Fig. 3B): the higher the GC-content, the more alternate molecules were returned, with the optimal GC content ranging between 45% and 75%. Probes with GC-content below 35% did not work well (Fig. 3C). This is very likely explained by the fact that low GC probes are longer (to match the required melting temperature) and generally more difficult to synthesize via solid-support method resulting in an inverse correlation between probe performance and length (Fig. 3D). Comparing different substitution classes for SNVs we only saw small differences in performance, with T > A variants having slightly lower efficiency compared to other variant types (Supplementary Fig. S5B). For InDels, different variant lengths had no significant impact on probe performance (Kruskal–Wallis test, two-sided, *P* = 0.52; Supplementary Fig. S5C). We also observed that the presence of an additional SNP at the 3′ end of the probe completely abolished probe performance (Supplementary Fig. S5D); explained by the specificity of pyrophosphorolysis whereby a mismatch blocks the polymerase from digesting the probe and releasing the targets. However, SNPs can be taken into account automatically during design of variant-specific probe pools and therefore are not problematic for Enspyre. Finally, we also identified several probes that performed better than expected (Fig. 3A), and we are currently investigating adding features into the probe model.

Overall, we confirmed that the probe design tool works well, and we are capable of creating a large variant-specific panel of probes without the need for testing or optimization.

### High selectivity of probes reduces the required depth of sequencing by ∼96%

Next, we calculated individual probe selectivity values for input VAFs higher than 0.1% (Fig. 3E). These values could be combined for different VAFs as the selectivity metric is independent of the input VAF (Supplementary Fig. S5E). On average, mean probe selectivity was 97 (median: 39) for Pool 1 and 120 (median 35) for Pool 2. Overall, 93.3% of probes in Pool 1 and 97.9% in Pool 2 enriched targeted variants (*S* > 1).

As shown in Table 1 most of the probes from both pools were either selective (10 < *S* < 50) or strongly selective (*S* > 50) for both InDels and SNVs. Most of the non-selective probes contained SNPs within 25 bp from the 3′-end of a probe (i.e. they were non-functional; Supplementary Fig. S5D) and could be improved in the future through the inclusion of these SNPs in the probe design. These, as well as probes targeting microsatellite (MS) InDels (see the next section), were removed from further analysis.

**Table 1.** Summary of estimated selectivity values for InDels and SNVs of both probe pools

	Pool 1		Pool 2	
Selectivity level	InDel	SNV	InDel	SNV
Nonselective: (0, 1]	3 (2.1%)	7 (0.49%)	4 (2.5%)	0 (0%)
Weakly selective: (1, 10]	12 (8.2%)	39 (2.7%)	13 (8%)	15 (1.0%)
Selective: (10, 50]	58 (40%)	823 (57%)	70 (44%)	984 (66%)
Strongly selective: >50×	73 (50%)	569 (40%)	74 (46%)	484 (33%)
Sum	146	1438	161	1483

Probes with overlapping SNPs and Indels in MS were removed. Selectivity level is presented as an interval for which lower and upper bounds are indicated. In parentheses next to the number of probes, we listed % of probes from the indicated Pool and variant type with a particular selectivity level, e.g. 823 SNV targeting probes from Pool 1 had a selectivity level between (10, 50], which is 57% out of all 1458 SNV targeting probes in Pool 1.

We then calculated the “panel wide” selectivity values (the harmonic mean of selectivity for all probes), which were 44.5 for Pool 1 and 31.0 for Pool 2. We used these values to estimate the reduction in sequencing depth needed for Enspyre to produce data equivalent to a non-selective assay. For example, for 1% VAF, we would reduce the sequencing depth needed by 96.8% for Pool 1 and 95.8% for Pool 2 compared to a non-selective assay, significantly lowering the cost of sequencing needed to identify rare variants.

### Enspyre can be used to detect single variants and predict input VAF

We next assessed the ability of Enspyre to detect individual variants. To detect individual variants by NGS, background error rates must be considered. These errors can be introduced during PCR cycles in the library preparation, the Enspyre protocol (via the hybridization buffer and through PCR), the sequencing itself or occasionally from low frequency somatic mutations in the cfDNA pool. Error rates are often mutation type-dependent [22] and should be higher in molecules for which a single strand has been sequenced (Supplementary Fig. S6A) than in “duplex” molecules for which both the forward and reverse strand were sequenced (Supplementary Fig. S6B), due to error correction by duplex consensus calling. The highest background error rates in this set of experiments were observed for InDels at homopolymer repeats or MS sites (Supplementary Fig. S6A and B). This is related to a phenomenon of DNA polymerase slippage at sites containing repetitive sequences [23, 24]. At present, Enspyre is analogous to other assays (e.g. Oncomine Dx Express test from Thermo Fisher Scientific) that are unable to analyse such sites. Consequently, all the results coming from probes targeting MS regions were removed from further analysis. For MRD detection, removing these variants is not a limitation due to the sufficient number of cancer-associated SNVs and non-MS InDels [6].

**Figure 4.**
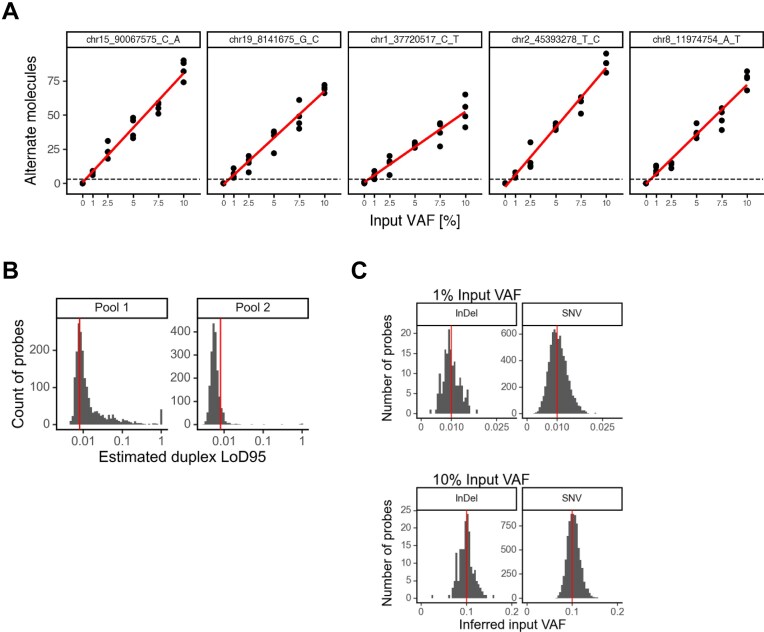
Enspyre can detect single variants and predict input VAF. (**A**) The number of targeted molecules recovered (i.e. the sum of duplex and non-duplex alternate molecules) generally increased linearly with the input VAF for individual probes (additional VAFs were tested to confirm linearity). The correlation of five randomly selected probes from Pool 2 is shown here. The differences between the slopes are due to the different probe performance (since all the VAFs are equal, the higher the slope value the better detection). (**B**) LoD95 values of variants determined by a regression-based analysis of the relationship of molecule counts at a target FPR of 2.84 × 10^−6^. Median LoD95 values at the FPR were ∼1% for probes of Pool 1 and ∼0.5% for probes of Pool 2. Probes with overlapping SNPs and Indels in MS were removed. The red line indicates the required LoD95 of the assay which we set at 0.8%. (**C**) The variant calling model we implemented also infers the original input VAF from the observed data. The distribution of the reconstructed input VAFs across variants is shown as histograms. The ground truth input VAF for the specific sample is highlighted by a vertical red line. Probes with overlapping SNPs and Indels in MS were removed. Data shown for Pool 2 only.

Overall, the data for individual probes in these experiments show a linear correlation with the input VAF (Fig. 4A for Pool 2, Supplementary Fig. S6C for Pool 1). From these data, we were able to estimate the input VAF at which we called 95% of samples (LoD95) with a threshold strict enough to meet the required FPR < 2.84 × 10^−6^ for each variant, corresponding to less than one expected FP variant call in 160 samples. Median LoD95 values at this FPR were ∼1% for probes in Pool 1 and ∼0.5% for probes in Pool 2 (Fig. 4B, Supplementary Fig. S6D, and Table 2).

**Table 2.** Summary of estimated LoD95 values for InDels and SNVs of Pool 1 and Pool 2

	Pool 1		Pool 2	
LoD level	InDels	SNVs	InDels	SNVs
(0, 0.5%]	1 (2.5%)	7 (0.5%)	22 (56.4%)	436 (29.4%)
(0.5%, 1%]	13 (32.5%)	701 (48.1%)	9 (23.1%)	1011 (68.1%)
(1%, 5%]	15 (37.5%)	552 (37.9%)	6 (15.4%)	32 (2.2%)
(5%, 10%]	5 (12.5%)	81 (5.6%)	0 (0.0%)	1 (0.1%)
(10%, 100%]	6 (15%)	117 (8.0%)	2 (5.1%)	4 (0.3%)
Total	40	1458	39	1484

Probes with overlapping SNPs and Indels in MS were removed. LoD level is presented as an interval for which lower and upper bounds are indicated. In parentheses next to the number of probes, we listed % of probes from the indicated Pool and variant type with a particular LoD level, e.g. 701 SNV targeting probes from Pool 1 had an LoD level between (0.5%, 1%] which is 48.1% out of all 1458 SNV targeting probes in Pool 1.

Additionally, due to the linearity between molecule counts and input VAF for individual probes (Fig. 4A), Enspyre data can also be used to reconstruct the input VAFs of variants. To this end, we used an experimental variant calling model for Enspyre taking into account known relationships between single-strand and duplex molecules for each probe as well as observed background error rates. With this model we were able to accurately recover input VAFs for Pool 2 from the data for individual variants (Supplementary Fig. S6E). For the samples with known 1% input VAF, the average and central 95% intervals of the inferred input VAFs were 1.00% (0.56%–1.53%) for InDels and 1.04% (0.52%–1.70%) for SNVs. For the 10% VAF group, the averages and central 95% intervals were 9.90% (7.16%–13.2%) for InDels and 10.3% (7.8%–13.4%) for SNVs.

In summary, Enspyre can be used to detect individual variants with an expected LoD95 of ∼0.5%. We also provide proof-of-principle data showing we can predict individual input VAFs.

### Enspyre can detect MRD down to a level of 0.01%–0.001% VAF

Recognizing MRD detection and patient monitoring as potential applications for the Enspyre technology, we used the data generated for 0.0001%–10% VAF contrived GIAB samples as well as 32 replicates for a LoB study (0%) to demonstrate that Enspyre can be applied to detect MRD.

**Figure 5.**
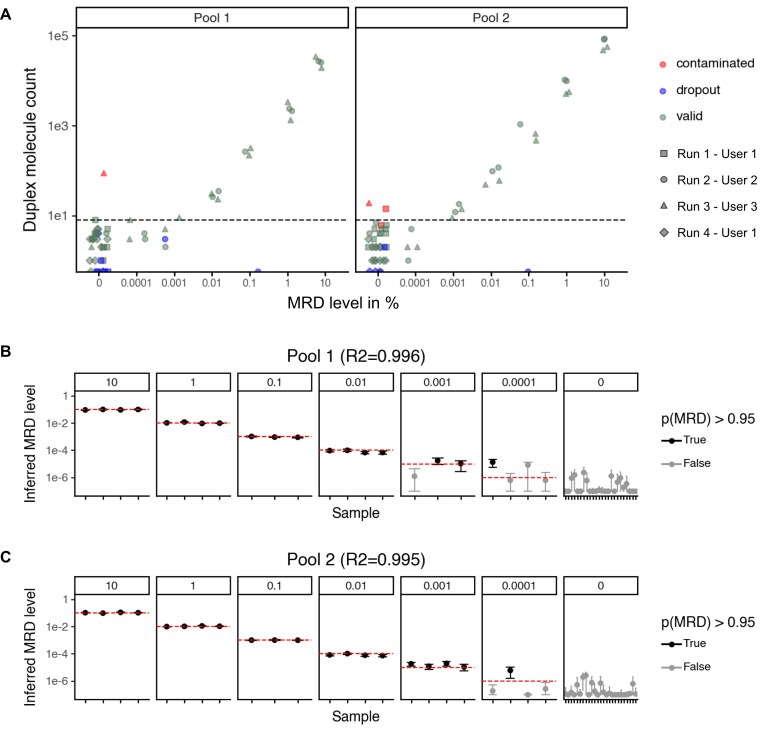
MRD detection using Enspyre data. (**A**) Analysis of the number of targeted variant molecules (duplexes) showed a clear relationship with the input DNA dilution (i.e. the MRD level). Datapoints from different runs are marked with different shapes on the plot (Run 1, 1st LoB; Run 2, 1st LoD; Run 3, 2nd LoD; and Run 4, 2nd LoB). All the runs were performed independently by indicated users. Samples that failed QC (dropouts) or were contaminated during sample preparation are shown in different colours. Blank samples (0% VAF) showed a background noise level below 8 molecules for both probe sets (dashed horizontal line). Molecule counts above the background level were observed for samples above 0.01% VAF (100 ppm) and increased continuously with the MRD input. (**B** and**C**) Model based analysis of all samples using Pool 1 (B) and Pool 2 (C) data demonstrate the ability to correctly identify MRD down to 0.01% VAF (100 ppm, Pool 1) and 0.001% VAF (10 ppm, Pool 2) without false-positive calls in the background blank samples. Red lines correspond to the known input VAF for each condition, e.g. for 100 000 ppm the MRD level is 0.1 (B and C).

Detection of MRD is limited by the level of noise coming from background errors and by the amount of data (i.e. number of variants available to track). As demonstrated previously, InDels in homopolymeric regions generally showed elevated background error rates (Supplementary Fig. S6A and B). Hence, we removed all InDel variants and only used SNVs to calculate a panel wide MRD score (Fig. 5A; Supplementary Fig. S7A and B).

The data were processed to determine the number of alternate molecules with a duplex sequencing consensus in each sample replicate. Overall, we observed background levels below 8 alternate duplex molecules across the 32 measured blank samples (0%) for both probe sets (see 0% group in Fig. 5A). Notably, for four samples we observed higher error levels (i.e. >8 alternate molecules). Upon further investigation, we found that these samples contained HG002 phased variants indicating accidental contamination with this DNA (see light grey dots in Fig. 5A). We then introduced experimental and environmental measures to mitigate the risk of cross-contamination (including separation of pre- and post-PCR areas during library preparation) and have not observed the problem since.

The remaining positive samples showed a clear increase in the alternate duplex molecule count above the background error level at 0.01% and 0.001% for the Pool 1 and Pool 2 probe set, respectively (Fig. 5A). The numbers of alternate molecules between four repeats for each MRD level were consistent despite the fact that they came from two independent runs performed by two different users. The samples above the lowest detected MRD level showed a linear increase of alternate duplex molecules with input. This linear relationship demonstrates that Enspyre could be used to quantitate the MRD level with a well selected probe set (such as Pool 2).

Finally, we also developed a statistical model to interpret Enspyre MRD data produced for any new probe set. This model is composed of two parts: a background error model based on observed error rates for different substitution types and a model describing the expected additional signal at different MRD levels for each probe. In short, the number of non-duplex and duplex calls for each probe is integrated across all probes to determine the probability of MRD and the MRD level per sample. Overall, this model was able to accurately quantitate the MRD level and detect the presence of MRD down to 0.01% for all samples using the Pool 1 probe set [*R*^2^ = 0.997 of log(MRD) for positive samples, Fig. 5B] and down to 0.001% for samples using the Pool 2 probe set [*R*^2^= 0.995 of log(MRD) for positive samples, Fig. 5C]. In both cases, no false-positives were observed for the LoB samples (i.e. observed specificity of 100%, 95% CI: 93.2%–100%).

Combined, these data provide proof-of-principle that Enspyre could be used in real-life applications such as MRD detection, achieving high sensitivity from a small fraction of the sequencing reads required with current approaches (Table 3). Further validation is needed to determine Enspyre’s sensitivity on clinical samples where mutations are present at different VAFs and to confirm reproducibility of the assay as only a limited number of repeats were included in the presented proof-of-principle experiments.

**Table 3.** A comparison of currently available MRD tests and their specifications

Test type	Company	Number of variants	Theoretical LoD (ppm)	Reported LoD (ppm)	Theoretical sequencing depth (Gb)
Tumour naïve	Grail	≥100 000	?	100–1000 [25]	12–15
Tumour informed	Natera	16	30	–	0.48
	NeoGenomics	48	10	∼10 [26]	1.44
	Haystack	50	10	–	1.5
	Invitae	50	10	80–500 [27]	1.5
	Personalis	1800	0.27	∼3.5 [6]	54
	Biofidelity	≥1800	≤0.27	≤10–100	≥2.27

The theoretical LoD of each assay assumes perfect detection and was calculated using 0.95 = 1 – poisson (0, tumour cell fraction * haploid genome copies * number of variants). The theoretical LoD of each assay is indicative of relative assay performance and is limited by sensitivity and specificity. The corresponding tumour cell fraction can be calculated using the number of variants in each assay and by assuming an input of 20 ng (∼6060 haploid genome copies). The reported LoD is the experimentally confirmed sensitivity for each of the tests. The theoretical sequencing depth was calculated as: number of variants*100 000 reads*300 nucleotides/1 000 000 000 with the following assumption: cfDNA is sequenced to a high depth of 100 000 reads using 2 × 150 bp paired end sequencing. There are no assumptions regarding on-target rates or variability of coverage. The sequencing depth of Enspyre is corrected for the “panel wide” enrichment of 23.8 (calculated from the “panel wide” selectivity of 31 for 1% input VAF).

## Discussion

Here, we present the Enspyre technology, a revolutionary modification to the traditional NGS hybridization capture methods, which enables selective enrichment of targeted variants. With its high efficiency and selectivity, Enspyre significantly reduces sequencing costs, analysis time and data storage, making identification of rare variants cheaper and faster. Importantly, similarly to other hybridization capture methods, we can also report individual input VAFs. Taken together, these qualities make Enspyre a suitable option for a variety of potential applications, from agriculture to diagnostics, including MRD detection for which the proof-of-concept is presented herein.

To our knowledge, the only other technology offering selective enrichment of rare variants similar to Enspyre is Maestro by Exact Sciences [28, 29], which uses short biotinylated oligos to discriminate between variants at the step of hybridization. At first glance, Maestro looks similar in terms of requirements (20 ng input into initial library preparation and 1 μg into hybridization compared to 20 ng and 900 ng for Enspyre, respectively). Maestro is more sensitive with reported LoD for single variants of 0.1% and MRD LoD of 1 ppm. However, this technology can only detect SNVs, currently does not produce individual variant input VAF estimates and is significantly more complex in a lab setting requiring preprocessing of oPools (including biotinylation and enzymatic digestion) as well as two rounds of hybridization and capture (each over 16 h). Double hybridization comes with more PCR rounds (32 versus 21 cycles for Enspyre) introducing more errors. Additionally, each sample needs to be fine-tuned for intrinsic noise. By comparison Enspyre, though not yet as sensitive, offers a simpler, more user-friendly option for rare variant enrichment.

In the technology proof-of-concept experiment we showed that Enspyre is capable of selectively targeting and enriching oncology-related variants by a mean of 35-fold compared to non-selective hybridization capture at a range of different input VAFs (0.1%–5%). This enrichment was dependent on the PPL reaction; without it, the Enspyre protocol performed as a non-selective assay.

We then developed the Enspyre probe design tool using an ML-based algorithm, which enabled the design of effective probes for specific variants without any testing or optimization. Average probe selectivity scores increased from 40 to 120, and nearly 98% of probes designed using the algorithm were selective. Some remaining probe design challenges include poor performance of AT-rich probes or those near an SNP. These could be mitigated by designing against the opposite strand for AT-rich targets, and by incorporating SNP information into the design step if available (e.g. for tumour-informed panels in cancer diagnostics). It should be noted that these current limitations only affect detection of individual variants and not sample-level detection of MRD status.

For individual variant detection, we estimate that Enspyre is able to achieve an LoD95 of 0.5% VAF, in line with other commercially available assays, but with lower DNA input and sequencing depth requirements. For example, the Illumina TruSight Oncology 500 ctDNA assay achieves an LoD95 of 0.5%, but using 50% higher input of ctDNA compared to Enspyre (see https://www.illumina.com/content/dam/illumina/gcs/assembled-assets/marketing-literature/trusight-oncology-500-ctdna-data-sheet-m-gl-00843/trusight-oncology-500-ctdna-data-sheet-m-gl-00843.pdf, accessed on 17 December 2024). Additionally, it also requires significantly higher sequencing depth produced by the NovaSeq 6000 System (output of 80–6000 Gb) compared to NextSeq 500 (output of 40 Gb) used in Enspyre. In principle, we could further increase sensitivity of Enspyre for individual variant detection by using higher sequencing depths.

For sample-level ctDNA detection, Enspyre was able to call MRD down to 0.01%–0.001% (10–100 ppm) based on 1800 interrogated variants and a total of 5 million read pairs per sample. Importantly, further validation is required to confirm MRD sensitivity of Enspyre with real clinical samples, where mutations are present at a variety of VAFs. The reproducibility of Enspyre also needs to be examined, as only a limited number of samples was interrogated for the proof-of-concept experiments presented here. By comparison, other MRD assays currently available on the market (Table 3) are either PCR-based and track a small number of variants limited by PCR multiplexing options (up to 50; e.g. Invitae Personalized Cancer Monitoring) or hybridization capture-based and track a higher number of variants (up to 1800; e.g. NeXT Personal from Personalis) but require ultra-deep sequencing (see Table 3). Due to its selective enrichment of variant molecules, Enspyre has the ability to combine the tracking of large panels of variants with a comparatively low sequencing depth, enabling implementation of sensitive MRD assays on benchtop NGS platforms. As such, variant enrichment technologies such as Enspyre and Maestro could remove access barriers to NGS-based MRD assays, which are currently too costly and complex to run for many clinical laboratories. Lower required sequencing depth also translates to reduced need for data storage, faster analysis and shorter turn-around times.

Additionally, Enspyre offers the flexibility to combine different tumour-type panels. In the MRD proof-of-concept experiments, we incorporated a fixed panel (nearly 4000 probes in total) that did not interfere with detection. Our preliminary data suggest that the number of probes could be increased even further without losing sensitivity. For the MRD application, this opens a possibility of increasing the number of tracked variants, potentially enabling detection below 0.001% MRD level. Finally, there is the opportunity to combine panels from different patients, simplifying the running of the assay by reducing the number of pools to synthesize, store, and handle.

With its capability of calling both individual variants as well as sample-level ctDNA presence, Enspyre’s potential clinical oncology applications range from therapy selection and tracking of tumour dynamics to treatment response monitoring and MRD detection. While further work is required to prove analytical and clinical validity of the assay, the proof-of-concept data described here demonstrate that Enspyre could become a useful new tool that has the potential to be a transformative technology for NGS-based assays.

## Supplementary Material

gkaf910_Supplemental_File

## Data Availability

All FASTQ files produced for this study are available at the NCBI Sequence Read Archive (https://www.ncbi.nlm.nih.gov/sra/) with Submission ID: SUB14820034, and Submission ID SUB15374175 (for the additional run data shown in Fig. 4A and Supplementary Fig. S7A). The binarized MRD code is publicly available at Zenodo together with output tables from performed analysis (https://doi.org/10.5281/zenodo.14392906).

## Supplementary data

Supplementary data is available at NAR online.

## Notes

Present address: Illumina Centre, Cambridge CB21 6DF, United Kingdom

Present address: AstraZeneca, Cambridge CB2 0AA, United Kingdom

Present address: Foresight Diagnostics, 2865 Wilderness Pl, CO 80301, United States

Present address: Bimodal, Cambridge CB10 1XL, United Kingdom
