# Supplementary material for: Enspyre: a novel enrichment technology for selected DNA variants using pyrophosphorolysis
Source: Nucleic Acids Res. 2025 Sep 17;53(17):gkaf910. doi: 10.1093/nar/gkaf910 (PMC12448872; doi:10.1093/nar/gkaf910)
Supplement: gkaf910_Supplemental_File [file gkaf910_supplemental_file.pdf]

SUPPLEMENTARY MATERIALS

Enspyre: A novel enrichment technology for selected DNA variants using pyrophosphorolysis

Katarzyna Anton[1]*, Timon Heide[1], Paulina Powalowska-Pickton[1], Ernesto Lowy-Gallego[1], Amy Lovell[1], Simonetta Andreazza[1], Efthimia Christoforou[1], Jeffrey Gregg[2], Sophie Hackinger[1], Magdalena Stolarek-Januszkiewicz[1], Robert J Osborne[1], Barnaby W Balmforth[1]

[1] Biofidelity Ltd, Cambridge, CB4 0WN, United Kingdom
[2] Biofidelity Inc., Morrisville, NC 27560, United States
*To whom correspondence should be addressed. Tel: +441223981750; Email: k.anton@biofidelity.com

Table of Contents

Supplementary Figure 1. Enspyre-independent PPL progression assay. 2
Supplementary Figure 2. Processing and analysis steps of NGS data. 3
Supplementary Figure 3. Variant selection for MRD proof of concept. 5
Supplementary Figure 4. Enspyre sequencing parameters. 6
Supplementary Figure 5. Additional probe features. 7
Supplementary Figure 6. Single variant detection – additional information. 8
Supplementary Figure 7. MRD detection – additional information. 10
Supplementary Methods 11

SUPPLEMENTARY FIGURE 1

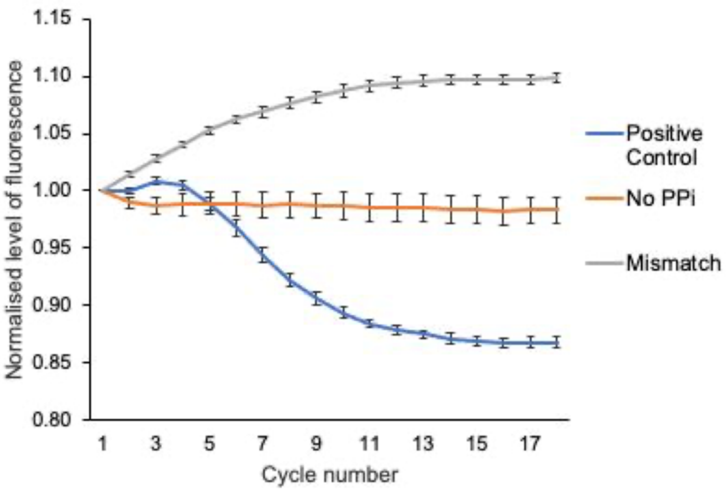

Supplementary Figure 1. Enspyre-independent PPL progression assay. PPL progression can be assessed via a qPCR-based Enspyre-independent assay in which probes hybridised with naked targets are subjected to PPL reaction. As PPL progressed, the fluorescence from an intercalating dye decreases, as seen in the positive control (in blue; probe fully complementary to the target in the presence of PPi). Without PPi, PPL does not progress even though probes and targets are complementary (orange). When a probe with a mismatch to the target is hybridised and subjected to PPL, reaction does not progress beyond the mismatch site (grey).

SUPPLEMENTARY FIGURE 2

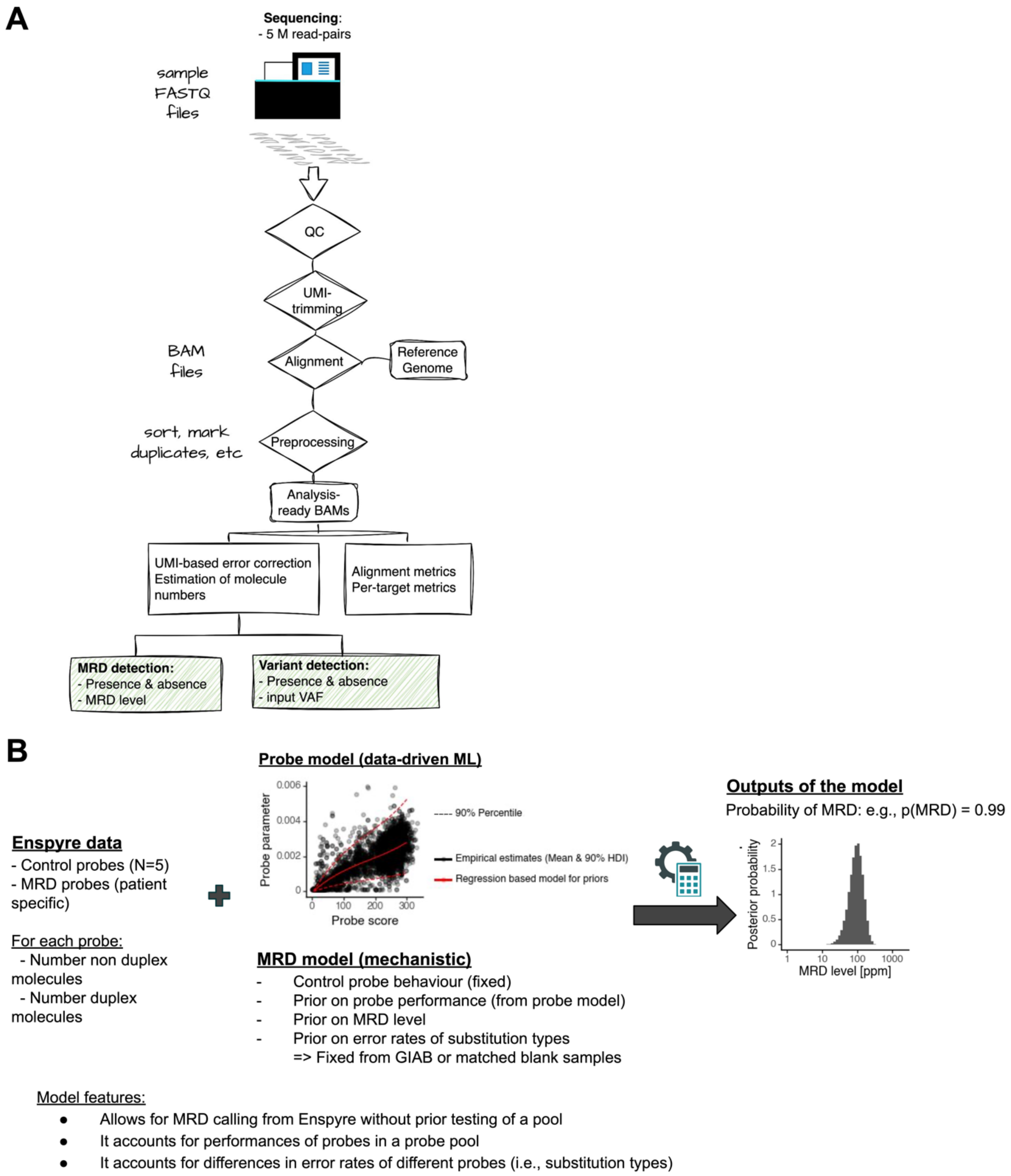

Supplementary Figure 2. Processing and analysis steps of NGS data. (**A**) Visual representation of the Enspyre analysis pipeline. Sequences are imported into the pipeline as FASTQ files and QC-ed. UMI-trimming is performed followed by alignment to the reference genome. Exported BAM files are preprocessed (sorted, duplicates marked, etc.). Alignment metrics and per-target metrics are checked. Next, UMI-based error correction is performed

followed by estimation of molecules numbers. Finally, individual variants are detected and input VAFs assessed. MRD detection model can be run at this stage. (**B**) Brief description of the MRD calling model. The input data required are: control probe data per run (assessing sample variability), MRD probe scores (from the probe design tool), numbers of duplex and non-duplex molecules retrieved. The data are fed into a mechanistic model of the assay. Within this model we estimate background error rates (based on observed error rates) and expected additional signal at different MRD levels for each probe. These data are then combined to produce probability of MRD per sample.

SUPPLEMENTARY FIGURE 3

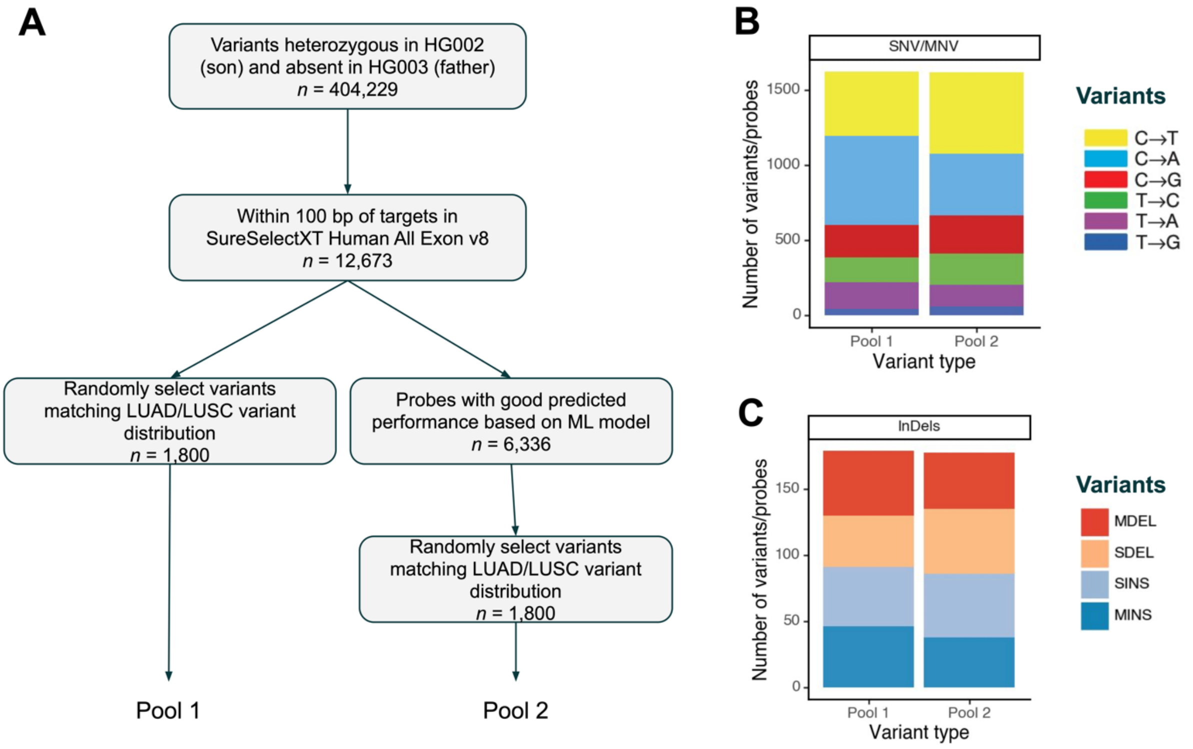

Supplementary Figure 3. Variant selection for MRD proof of concept. (**A**) Variants heterozygous in HG002 (1/0, son) and absent from HG003 (0/0, father) were identified (404,229). Variants within 100 bp of the targets from the SureSelectXT Human All Exon v8 panel were filtered to produce a final set of 12,673 variants for probe design. For Pool 2, variants were further filtered to only include efficient probes based on the ML scoring (6,336). For both probesets, probes were designed against randomly sampled variants matching the distribution of somatic variants in lung adenocarcinoma (LUAD) and lung squamous cell carcinoma (LUSC). (**B**) The distribution of substitution classes in the two generated probesets. (**C**) The distribution of deletion types in the two generated probesets, including: multiple base deletion (MDEL), single base deletion (SDEL), single base insertion (SINS), multiple base insertion (MINS).

SUPPLEMENTARY FIGURE 4

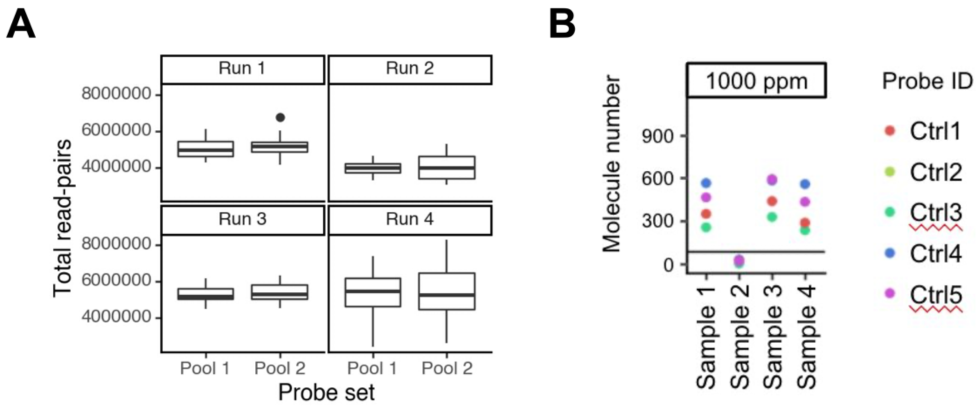

Supplementary Figure 4. Enspyre sequencing parameters. (**A**) Following Enspyre, libraries can be sequenced with relatively low sequencing depth, e.g. for this study we used Illumina NextSeq500 with a mid output kit for 2x150 cycles to produce paired-end reads. The total number of generated read-pairs per sample were just under 5 M per sample for both probe sets, with some variability across different sequencing runs. Run1 - 1st limit of blank, Run2 - 1st limit of detection, Run3 - 2nd limit of detection, Run4 - 2nd limit of blank. (**B**) Enspyre control probes return a number of molecules per variant representative of the sample quality. If a number of molecules per probe is lower than 100 for at least one of the control probes, sample is marked as failed (e.g. Sample 2).

SUPPLEMENTARY FIGURE 5

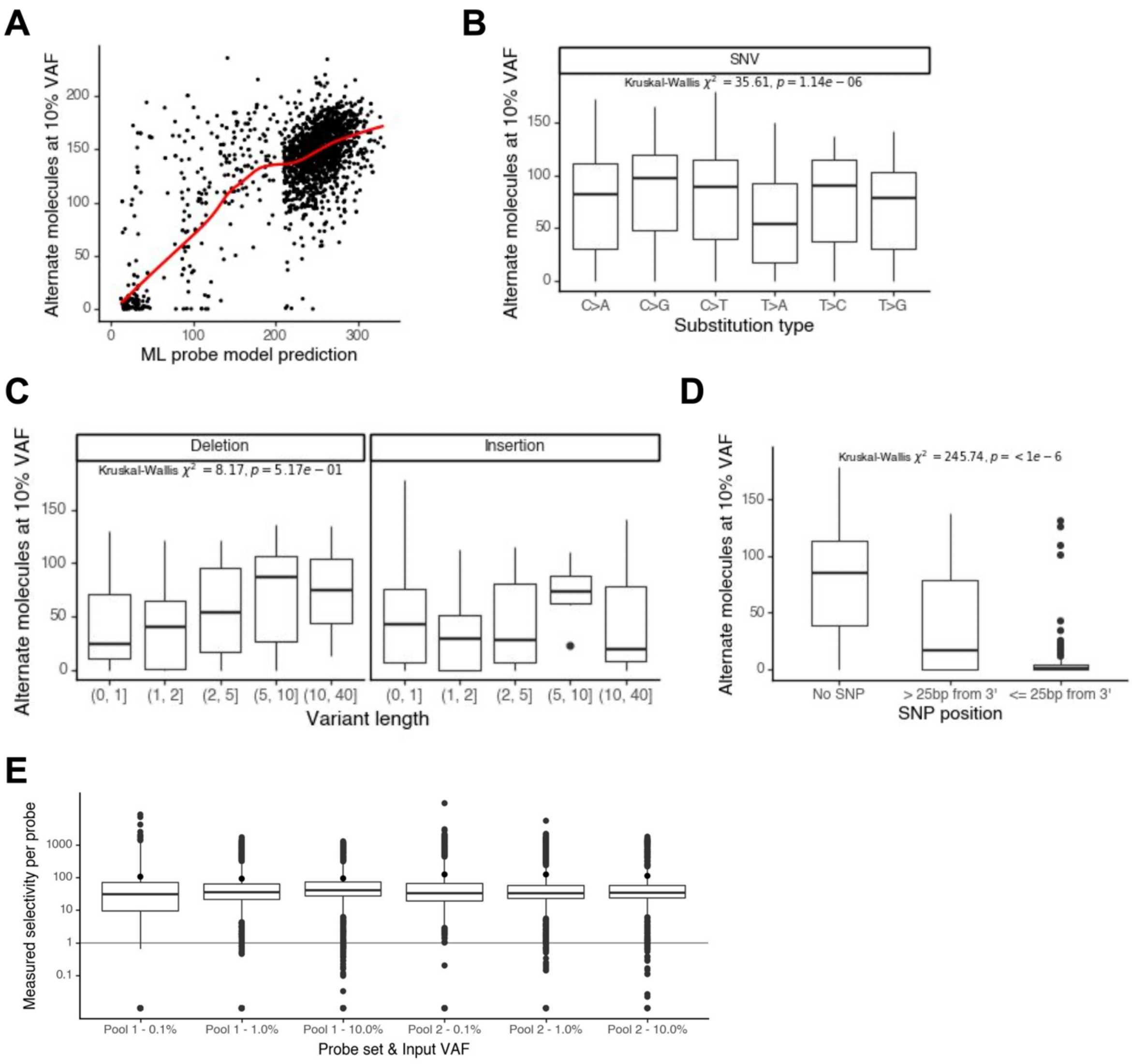

Supplementary Figure 5. Additional probe features. (**A**) Targeted variant molecule recovery of each probe in Pool 2 at 10% input VAF plotted against the probe design algorithm scores. Majority of probes recovers high numbers of alternate molecules due to additional filtering based on predicted ML probes model scores. (**B**) There are small, but significant differences between different substitution types (SNVs) and the efficiency of associated probes (Kruskal-Wallis test, two-sided, $p < 0.0001$). (**C**) There are no significant correlations between the InDel type (i.e., deletion or insertion) and the length of these variants. (**D**) Probes with a SNP in the first 25 base pairs of the probe sequence are generally non-functional. (**E**) Selectivity metric for Pool 1 and Pool 2 across a range of different input VAFs. All the boxplots encompass lower and upper quartile of the data with the highlighted median value.

SUPPLEMENTARY FIGURE 6

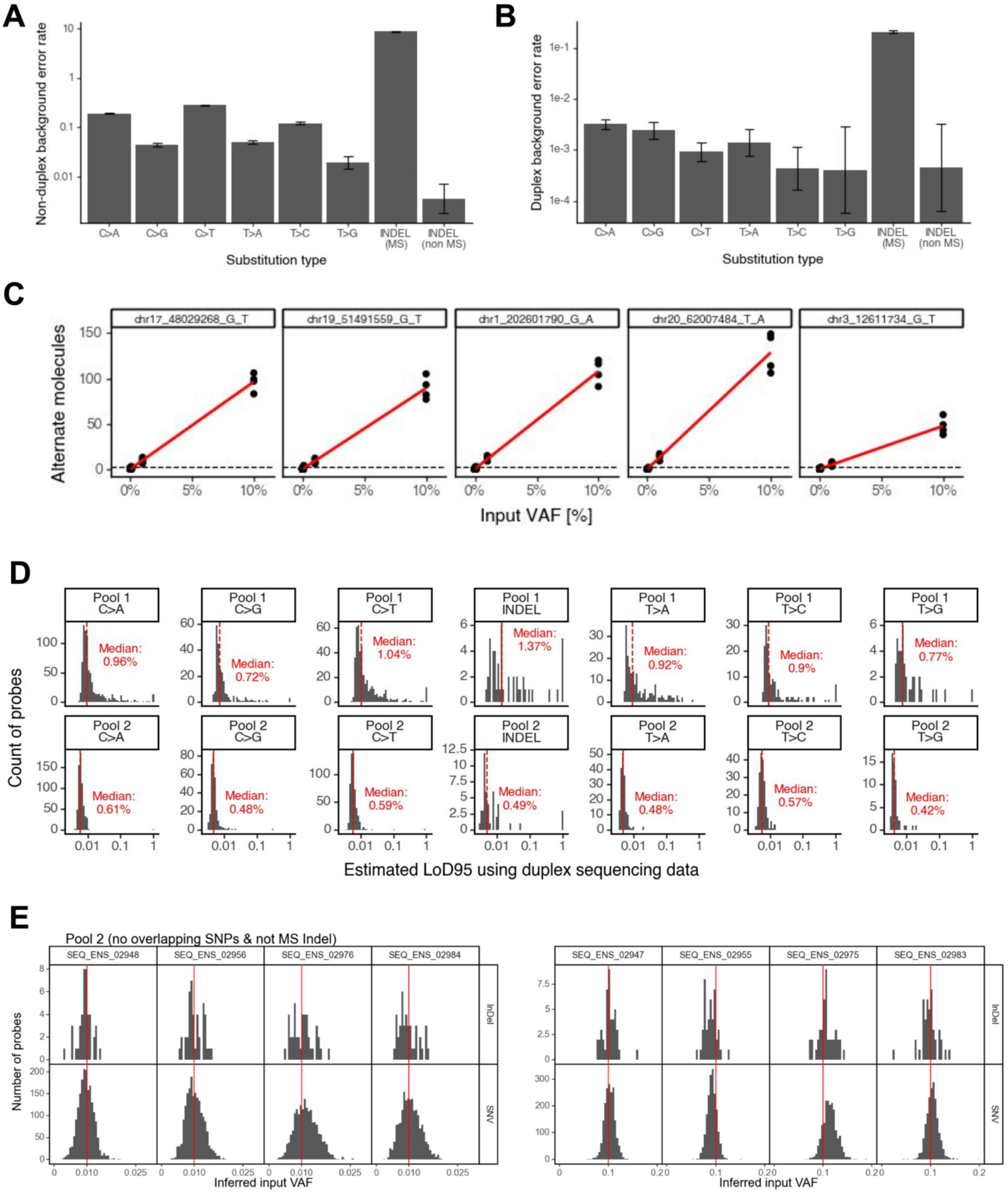

Supplementary Figure 6. Single variant detection - additional information. (**A**) Different substitution types showed variation in background error rates (mean number of off-target molecules recovered from blank samples). Especially, InDels in homopolymers or microsatellites (MS) showed very high error rates that likely result from polymerase slippage. The background error rates of InDels not in microsatellites (non MS) were lower than all

other variant types. (**B**) Similar to the single-strand consensus molecules, double strand consensus molecules (i.e., duplex molecules) showed variation of background error rates for different substitution types. (**C**) The number of targeted molecules recovered (i.e., the sum of duplex and non-duplex alternate molecules) generally increased linearly with the input VAF for individual probes. The correlation of five randomly selected probes from Pool 1 is shown here. The differences between the slopes are due to the different probe performance (since all the VAFs are equal here, the higher the slope value the better detection). (**D**) LoD values vary according to the mutation type. LoD95 values of variants were determined by a regression-based analysis of the relationship of molecule counts at a target false-positive rate (FPR) of $2.84 \cdot 10^{-6}$. Probes with overlapping SNPs and Indels in MS were removed. (**E**) The distribution of the reconstructed input VAFs across variants split by variant type (InDel vs SNV) and sample is shown as histograms. The ground truth input VAF for the specific sample is highlighted by a vertical red line. Probes with overlapping SNPs and Indels in MS were removed.

SUPPLEMENTARY FIGURE 7

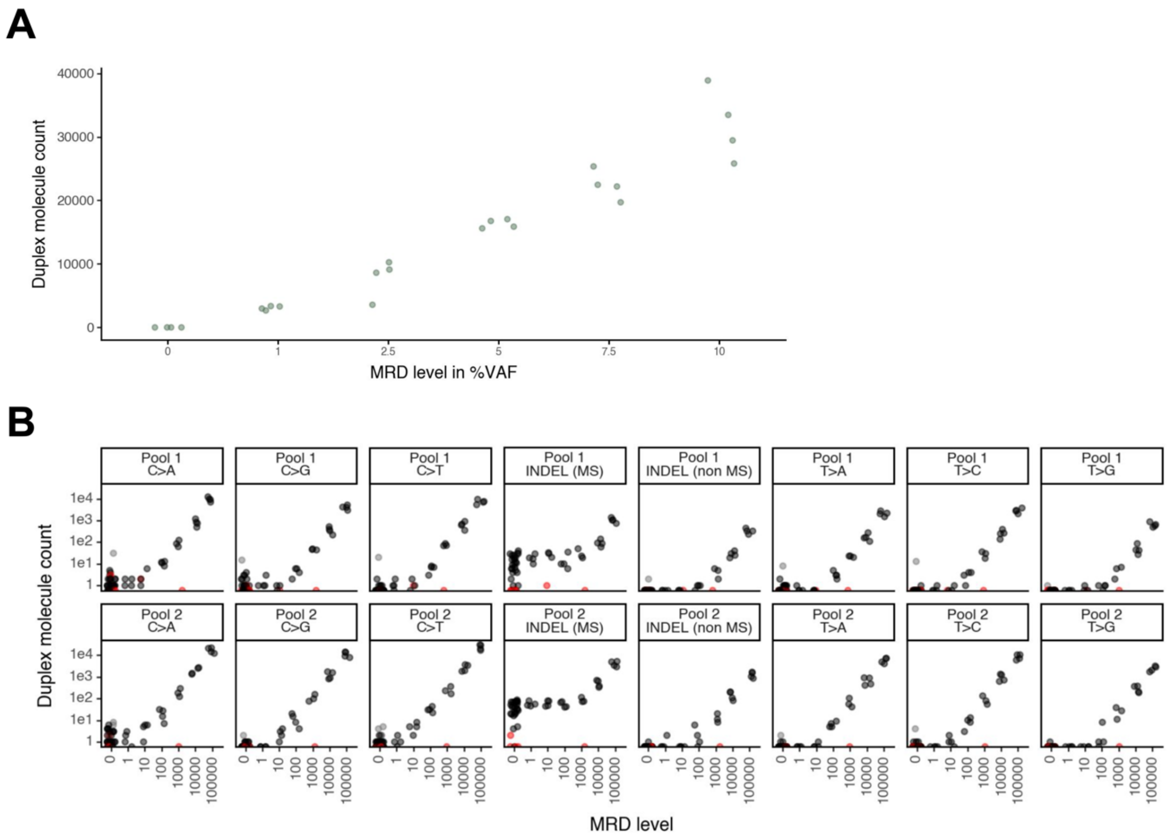

Supplementary Figure 7. MRD detection - additional information. (**A**) Analysis of the number of targeted variant molecules (duplexes) showed a clear relationship with the input DNA dilution (i.e., the MRD level) in an additional experiment with intermediate VAF levels (0, 1, 2.5, 5, 7.5 and 10%). (**B**) MRD detection works well for all the mutation types apart from MS InDels. Targeted variant duplex molecule count split by probe pool (1 or 2) and by mutation type. MS InDels (in homopolymeric regions) showed elevated background error rates making these types of mutation unsuitable for MRD detection via Enspyre.

Supplementary Methods

Enspyre-independent PPL assay

The assay was performed in 10 µL PPL mix with or without PPi (commercially available Biofidelity Ltd) supplemented with Apyrase (2 U/mL,New England Biolabs, M0398B) and EvaGreen (1x, Cambridge Biosciences, BT31000) on 250 nM T790M target with either fully complementary T790M probe or T790M mismatched probe (custom synthesis by IDT). Reactions were set up on ice, transferred to a 384-well plate (Thermo Scientific, AB3384/B) and run on CFX384 Touch Real-Time PCR Detection System (BioRad) at constant temperature of 45°C and images taken every minute for at least 25 min in total.

The following oligos were individually ordered from IDT for this experiment:

1) T790M biotinylated Probe:
   /5Biosg/TTTTTTTTTTTTTTTTTTTTCCAGGAGGCAGCCGAAGGGCATGAGCTGC**A**TGATG
2) MUT EGFR T790M target (Positive control):
   CTGCTGGGCATCTGCCTCACCTCCACCGTGCAGCTCATCA**T**GCAGCTCATGCCCTTCGGCTGCCTCCTGGACTATGTCCGG
3) WT EGFR Exon20A target (Mismatch):
   AATGATACGGCGACCACCGAGATCTACACTGGTAATTACCGACGAAAACGGCCCGTGCAGCTCATCA**C**GCAGCTCATGCCCTTCGGCTGCCTCCTGGACTATGTCCGGGAACCGCAAGACTGTAACCACGCGTATCTCGTATGCCGTCTTCTGCTTG
